# Airway administration of bisphosphate and dexamethasone inhibits SARS-CoV-2 variant infection by targeting alveolar macrophages

**DOI:** 10.1038/s41392-022-00977-1

**Published:** 2022-04-06

**Authors:** Zhenfeng Wang, Yabo Zhou, Linlin Bao, Dan Li, Jiadi Lv, Dianheng Wang, Shunshun Li, Jiangning Liu, Chuan Qin, Wei-Min Tong, Bo Huang

**Affiliations:** 1 Department of Immunology & National Key Laboratory of Medical Molecular Biology, Institute of Basic Medical Sciences, Chinese Academy of Medical Sciences (CAMS) & Peking Union Medical College, 100005 Beijing, China; 2 NHC Key Laboratory of Human Disease Comparative Medicine, Beijing Key Laboratory for Animal Models of Emerging and Remerging Infectious Diseases, Institute of Laboratory Animal Science, CAMS and Comparative Medicine Center, Peking Union Medical College, Beijing, China; 3 Department of Pathology, Institute of Basic Medical Sciences, CAMS and Peking Union Medical College, Beijing, China; 4 Department of Biochemistry & Molecular Biology, Tongji Medical College, Huazhong University of Science & Technology, 430030 Wuhan, China

**Keywords:** Innate immune cells, Drug development, Infection

**Dear Editor**,

Alveolar macrophages (AMs) are among the first immune cells to encounter SARS-CoV-2 during an infection due to their abundant numbers and physical location in the lungs. Thus, the reaction of AMs to SARS-CoV-2 has a profound impact on the outcome of the infection. In most cases, AMs can release cytokines and prime adaptive T- and B-cell immune responses to resolve the infection. However, in some cases, the accumulation of SARS-CoV-2-infected macrophages in the lungs is associated with severe lung pathology and poor prognosis of the disease.^1^ The latter observation has been interpreted as the result of excessive cytokine production by pro-inflammatory macrophages that induce protracted local and systemic uncontrolled inflammatory responses.^2^ However, it remains unclear if infected AMs will limit viral replication or serve as a viral reservoir to produce de novo infectious viral particles.

Our recent studies have demonstrated that the fate of SARS-CoV-2 in infected AMs is polarization-dependent where viral replication is permissive in M1 AMs but restricted in M2 AMs. We found that anti-inflammatory M2 AMs have a higher endosomal pH than pro-inflammatory M1 AMs, thus limiting SARS-CoV-2 release from endosomes and leading to viral degradation in lysosomes.^3,4^ This process is mediated by endosomal protease cathepsin L (CTSL) that cleaves SARS-CoV-2 spike protein in a low pH environment, allowing the viral and endosomal membrane fusion and the subsequent release of viral RNA into the cytoplasm of macrophages. This endosomal restriction of M2 macrophages, however, may be broken through by Delta variant, which uses spike protein mutations to relieve the inhibition of the relatively high endosomal pH on CTSL.^5^ Thus, targeting CTSL and endosomal pH of AMs might be a potential strategy to curb SARS-CoV-2 infection at the initial stage.

Bisphosphate such as alendronate (ALN) is widely used in osteoporosis treatment by targeting macrophages such as osteoclasts. Upon the uptake by macrophages, ALN forms an ATP analog, thus interfering with ATP homeostasis, which may indirectly influence endosomal V-ATPase and H^+^ concentration. In addition, dexamethasone (Dex), a glucocorticoid drug, has already been used to inhibit the pathological inflammation in SARS-CoV-2-infected patients.^6^ Notably, previous studies have indicated that M1 macrophages express higher levels of CTSL than M2 macrophages^7^ and Dex can polarize macrophages into M2 phenotype. Given the influence on phenotype of AMs, we speculated that Dex might also regulate the CTSL expression. We thus tested the possibility of that ALN combining with Dex prevented SARS-CoV-2 infection by targeting the CTSL-endosomal pH axis of AMs. Meanwhile, considering the alveolar surfactant barrier, which prevents the systemic drug molecules from entering the alveoli, a local inhalation was practiced for this combination.

In this study, we first determined CTSL expression in M1 and M2 AMs. The result showed that a higher expression of CTSL was observed in M1 rather than M2 macrophages (Supplementary Fig. S1a, b). Then, we used Dex (0.2 μM or 1 μM) and ALN (10 μM or 50 μM) to treat AMs in vitro. We found that either low or high dosage of Dex was able to downregulate CTSL expression, and ALN with the 50 μM dosage increased endosomal pH (Fig. 1a, b), implying that Dex and ALN are potential agents in intervening SARS-CoV-2 infection of AMs. Following 2 h infection of Delta variant, AMs were treated with Dex, ALN, or both for 22 or 46 h. The viral load, reflected by the expression of viral NP and ORF1ab, was reduced in single ALN, Dex, or combination group (Fig. 1c; Supplementary Fig. S1c). We further used ACE2-overexpressing A549 cells to verify this result. Consistently, the combination of Dex and ALN effectively inhibited the viral replication (Fig. 1d; Supplementary Fig. S1d). In line with these in vitro results, in the intranasally treated mice, we found that Dex indeed downregulated CTSL expression in AMs (Fig. 1e) and ALN increased the endosomal pH (Fig. 1f).

**Fig. 1:**
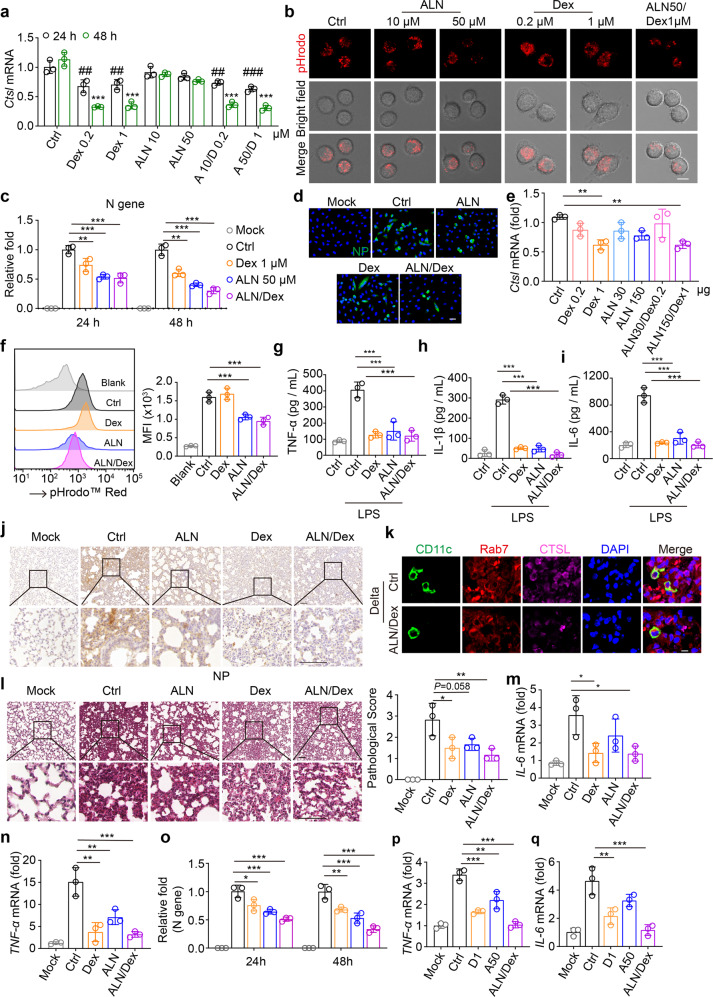
Combination of Dex and ALN is able to treat SARS-CoV-2 Delta variant infection. **a** AMs isolated from ICR mice were treated with Dex, ALN or combination for 24 h or 48 h. The *Ctsl* mRNA was analyzed by qPCR. D, Dex; A, ALN. “#” and “*” are compared with “24 h-Ctrl” and “48 h-Ctrl” group, respectively. **b** AMs isolated from ICR mice were treated with Dex, ALN or combination for 24 h, stained with pHrodo™ Red dextran (red), and observed under confocal microscope. Scale bar, 10 μm. **c** AMs isolated from ICR mice were infected with SARS-CoV-2 Delta variant (2.5 × 10^4^ TCID50) for 2 h, then virus was removed and cells were re-cultured with dexamethasone, alendronate or combination for another 22 h or 46 h. The level of virus load was analyzed by qPCR. **d** ACE2-overexpression A549 cells were incubated with Delta for 4 h, then virus was removed and cells were re-cultured with dexamethasone (1 μM), alendronate (50 μM) or combination for another 20 h. Cells were stained with anti-NP antibodies. Scale bar, 20 μm. **e**, **f** Dex, ALN or both were delivered to ICR mice by intranasal administration. Twenty-four hours later, AMs were collected to validate *Ctsl* expression (**e**) or endosomal pH analysis (**f**). **g**–**i** ICR mice were treated with LPS (100 μg/per mouse) by intranasal administration. Four hours later, dexamethasone (1 μg/per mouse) or alendronate (150 μg/per mouse) or both were given. At 24 h, the level of TNF-α (**g**), IL-1β (**h**) or IL-6 (**i**) in bronchoalveolar lavage fluid (BALF) were analyzed by ELISA. **j**–**n** hACE2-transgenic mice were infected with 1 × 10^5^ TCID_50_ Delta. At 2 h post infection, administered with Dex (i.n., 50 μl, 1 μg), ALN (i.n., 50 μl, 150 μg) or both once per day for 5 days. The lung tissues were fixed for NP (**j**) and CTSL immunostaining (**k**) or H&E staining (**l**). The mRNA of *IL-6* (**m**) and *TNF-α* (**n**) were detected by qPCR. **k** scale bar, 10 μm. **l** scale bar, 100 μm. Rab7, endosomal marker; CD11c, a marker of AMs. **o**–**q** AMs isolated from ICR mice were infected with SARS-CoV-2 Omicron variant (2.5 × 10^4^ TCID50) for 2 h, then virus was removed and cells were re-cultured with dexamethasone, alendronate or combination for another 22 h or 46 h. The level of virus load (**o**), TNF-α (**p**) and IL-6 (**q**) was analyzed by qPCR. The data represent mean ± SD. * *p* < 0.05, ** *p* < 0.01, *** *p* < 0.001, by one-way ANOVA (**a**, **c**, **e**–**i**, **l**–**q**)

Upon SARS-CoV-2 infection, alveolar macrophages upregulate pro-inflammatory cytokines, which may exacerbate the pulmonary pathology. In line with this, Delta variant strongly stimulated AMs to upregulate the expression of TNF-α, IL-1β, and IL-6, which however could be abrogated by Dex and ALN (Supplementary Fig. S1e–g). To further validate this in vitro result in vivo, we used lipopolysaccharide (LPS) to treat mice by airway administration, which recapitulates aspects of the inflammatory cascades associated with pulmonary inflammation. Following 4 h effect by LPS, we intranasally treated the mice with Dex, ALN, or both. Twenty-four hours later, we found that the expression of TNF-α, IL-1β, and IL-6 was downregulated in AMs by the treatment (Supplementary Fig. S1h–j). Moreover, the ELISA analysis showed that the levels of IL-1β, TNF-α, and IL-6 were also reduced in the bronchoalveolar lavage fluids (Fig. 1g–i).

Finally, we translated the above findings in the Delta variant-infected mouse model. The hACE2-transgenic mice were infected with Delta variant for 2 h, followed by the intranasal treatment of Dex, ALN, or both once per day for 5 days. We found that either Dex or ALN alone had the inhibitory effect on NP expression at the mRNA and protein levels, and the combination generated highest inhibition (Fig. 1j; Supplementary Fig. S1k). Consistently, ALN/Dex treatment resulted in the downregulation of CTSL in the endosomes of macrophages (Fig. 1k). The H&E staining also showed that the combination of Dex and ALN led to the minimum pathological damage in the lungs (Fig. 1l). In line with these results, the combination markedly decreased the expression of TNF-α and IL-6 at both mRNA and protein levels in the lungs of the infected mice (Fig. 1m, n; Supplementary Fig. S1l, m).

Apart from the above Delta variant, here we also tested Omicron variant, the current dominant variant worldwide. First, we compared the cleavage of S protein from Delta and Omicron by CTSL under different pH conditions. We found that under a pH 6.0 or 6.5 condition, CTSL more strongly cleaved Delta S protein than Omicron S protein (Supplementary Fig. S1n), implying that Dex/ALN treatment also interferes with CTSL to cleave Omicron S protein. In this regard, we infected AMs with Omicron variant for 2 h, followed by the treatment of Dex, ALN, or both for 22 or 46 h. As expected, the viral load, reflected by viral NP and ORF1ab, was reduced by Dex/ALN (Fig. 1o; Supplementary Fig. S1o). On the other hand, the pro-inflammatory cytokines were also downregulated in the infected cells by Dex/ALN (Fig. 1p, q; Supplementary Fig. S1p). These results, together our previous studies,^5^ suggest that Dex/ALN may be used to treat different SARS-CoV-2 variant.

Vaccines and antiviral drugs are currently two major arms, which greatly prevent SARS-CoV-2 spread. However, vaccines are unable to treat the already infected patients and may become invalid to viral mutants. On the other hand, antiviral drugs cannot be used as a prophylactic and screening small compound(s) to target viral life cycle is facing non-specificity and unsafety. Our present study provides evidence that AMs as the first immune cells can become a useful target to curb the spread of SARS-CoV-2 including the variants.

Majority of people have no symptoms or just have mild ones following SARS-CoV-2 infection. This fact suggests that the human immune system can clear SARS-CoV-2 infection at an early stage, implying a pivotal role of AMs in this process. This is because that the virus mainly invades the alveoli, the air sac structure located at the end of bronchioles, where ~95% resident immune cells are macrophages.^3^ Histologically, the internal surface of the alveoli is lined with the alveolar subphase fluid and pulmonary surfactant. Such subphase fluid and surfactant are derived from type I and type II alveolar epithelial cells, respectively, thus only allowing AMs rather than alveolar epithelial cells to be contacted by alveolus-inhaled SARS-CoV-2. Following the uptake by AMs, the virus faces two fates: being either delivered to the lysosome for degradation or released to the cytosol for viral proliferation. We demonstrate that SARS-CoV-2 is destined to the first way in AMs by the combination of two old drugs, Dex and ALN. This is because Dex and ALN can complementarily interfere with the endosomal release pathway by targeting CTSL expression and endosomal pH, respectively.

Local delivery of drugs via airway is pivotal to achieve the early intervention. The conventional systemic administration may face the separation of the alveolar surfactant, thus impeding systemic Dex and ALN to gain access to AMs. Indeed, the nasal rather than the intravenous pre-administration of Dex and ALN was able to effectively inhibit LPS-induced pro-inflammatory cytokine expression and to increase endosomal pH in AMs (Supplementary Fig. S1q, r). Relative to the decrease of CTSL by Dex, the increase of endosomal pH by ALN seems to have more impact on viral load, while the effect of Dex on inflammation inhibition is better than that of ALN, leading to an ideal combination strategy against SARS-CoV-2 variant infection. All in all, in addition to suppressing inflammation, alveolar administration of Dex and ALN can prevent the release of SARS-CoV-2 RNA from endosomes into the cytosol by increasing endosomal pH of AMs, thus facilitating the degradation of the virus in lysosomes and promoting the early control of the viral infection in a simple, safe, and low-cost way.

## Supplementary information

supplementary information

## Data Availability

All data needed to evaluate the conclusions in the paper are present in the paper or the Supplementary Materials. Materials described in the study are either commercially available or on request from the corresponding author.
