# Supplementary material for: Airway administration of bisphosphate and dexamethasone inhibits SARS-CoV-2 variant infection by targeting alveolar macrophages
Source: Signal Transduct Target Ther. 2022 Apr 6;7:116. doi: 10.1038/s41392-022-00977-1 (PMC8984664; doi:10.1038/s41392-022-00977-1)
Supplement: Supplementary file 1 — supplementary information [file 41392_2022_977_MOESM1_ESM.docx]

**Supplementary Information**

Airway administration of bisphosphate and dexamethasone inhibits SARS-CoV-2 infection by targeting alveolar macrophages

Zhenfeng Wang^1,5^, Yabo Zhou^1,5^, Linlin Bao^2^, Dan Li^2^, Jiadi Lv^1^, Dianheng Wang^1^, Shunshun Li^1^, Jiangning Liu^2^, Chuan Qin^2*^, Wei-Min Tong^3*^, Bo Huang^1,4*^

^1^Department of Immunology & National Key Laboratory of Medical Molecular Biology, Institute of Basic Medical Sciences, Chinese Academy of Medical Sciences (CAMS) & Peking Union Medical College, Beijing 100005, China

^2^NHC Key Laboratory of Human Disease Comparative Medicine, Beijing Key Laboratory for Animal Models of Emerging and Remerging Infectious Diseases, Institute of Laboratory Animal Science, CAMS and Comparative Medicine Center, Peking Union Medical College, Beijing, China.

^3^Department of Pathology, Institute of Basic Medical Sciences, CAMS and Peking Union Medical College, Beijing, China

^4^Department of Biochemistry & Molecular Biology, Tongji Medical College, Huazhong University of Science & Technology, Wuhan 430030, China

^5^These authors contributed equally

*Corresponding author:

[tjhuangbo@hotmail.com](mailto:tjhuangbo@hotmail.com), [Weiminfr@hotmail.com](mailto:Weiminfr@hotmail.com), [qinchuan@pumc.edu.cn](mailto:qinchuan@pumc.edu.cn)

**This PDF file includes:**

Figure. S1

Materials and Methods


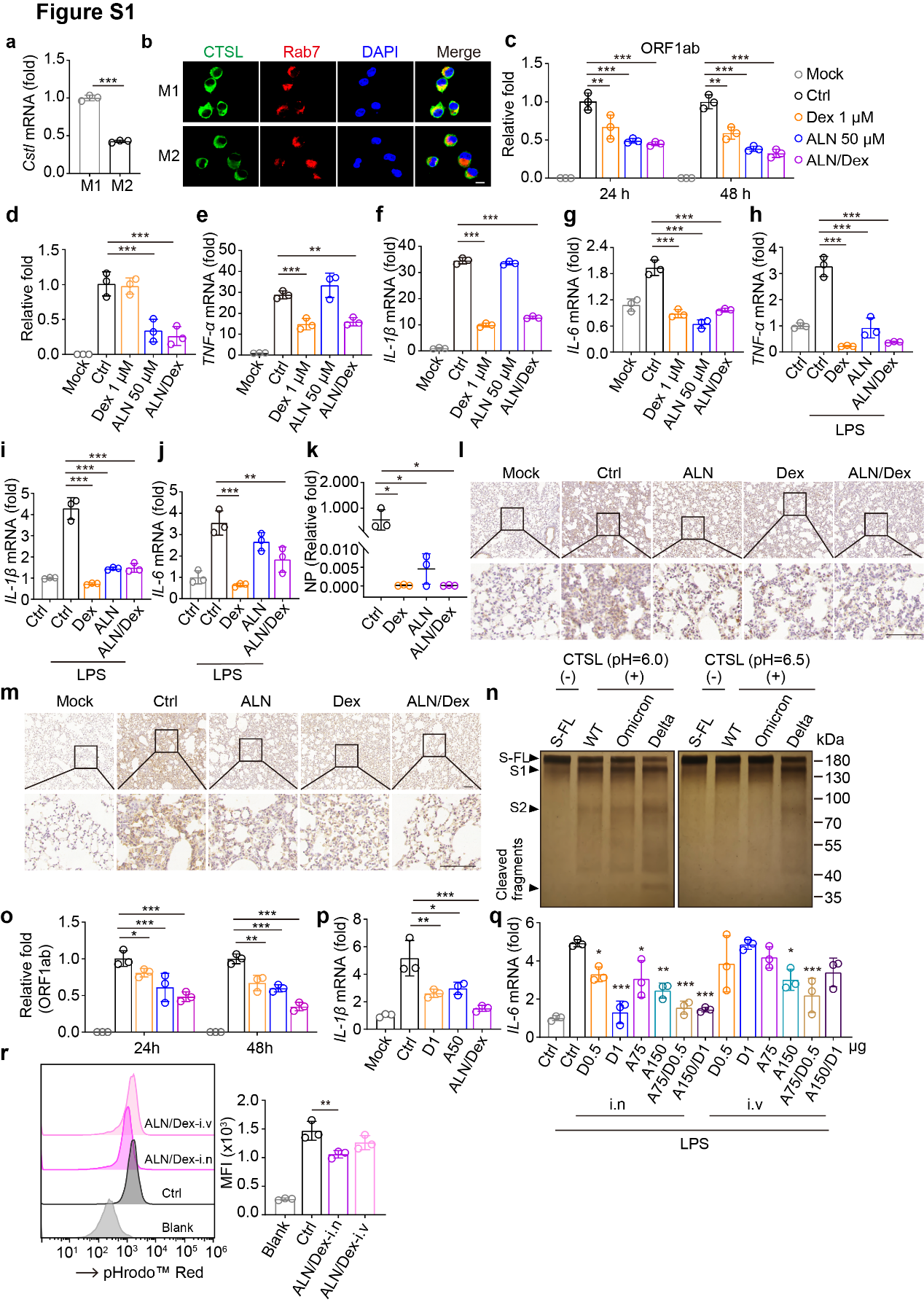


**Fig. S1 Combination of Dex and ALN could attenuate viral load and cytokines release. a, b,** AMs were isolated from ICR mice. M1 AMs were induced by LPS (100 ng/mL) and IFN-γ (20 ng/mL) or M2 were generated by IL-4 (20 ng/mL). Then cells were used for real-time PCR analysis (a) and immunofluorescent staining of rab7 and CTSL (b). Rab7, a marker of endosomes. Scale bar, 10 μm. **c,** AMs isolated from ICR mice were infected with SARS-CoV-2 Delta variant (2.5×10^4 TCID_50_) for 2 h, then virus was removed and cells were re-cultured with dexamethasone, alendronate or combination for another 22 h or 46 h. the level of virus load was analyzed by qPCR. **d,** ACE2-overexpression A549 cells were incubated with Delta for 4 h, then virus was removed and cells were re-cultured with dexamethasone (1 μM), alendronate (50 μM) or combination for another 20 h. Virus load (N gene) was analyzed by qPCR. **e-g,** The same as (c), except that the expression of TNF-α (e), IL-1β (f) or IL-6 (g) were analyzed by qPCR. **h-j,** ICR mice were treated with LPS (100 μg / per mouse) by intranasal administration. 4h later, dexamethasone (1 ug / per mouse) or alendronate (150 ug / per mouse) or both were given. At 24 h, the level of TNF-α (h), IL-1β (i) or IL-6 (j) in AMs were validated by qPCR. **k-m,** hACE2-transgenic mice were infected with 1×10^5^ TCID_50_ Delta. At 2 h post infection, administered with Dex (i.n., 50 μl, 1 μg), ALN (i.n., 50 μl, 150 μg) or both once per day for 5 days. The virus load in lung tissues was detected by qPCR (k) and the tissues were fixed for TNF-α (l) or IL-6 immunostaining (m). Scale bar, 100 μm. **n,** Comparison of CTSL cleavage efficiency among different variants. The cleavage of spike protein (1 μg) by CTSL (20 ng) at 37℃ for 5 min is visualized by silver staining. Black arrow heads indicate spike protein bands. **o-p,** AMs isolated from ICR mice were infected with SARS-CoV-2 Omicron variant (2.5×10^4 TCID_50_) for 2 h, then virus was removed and cells were re-cultured with dexamethasone, alendronate or combination for another 22 h or 46 h. the level of virus load (o) and IL-1β (p) was analyzed by qPCR. **q,** Dex, ALN or both were delivered to ICR mice by intranasal or intravenous administration. 2 h later, LPS was given and AMs were collected at 4 h post drug treatment. The IL-6 expression was determined by qPCR. **r,** Dex, ALN or both were delivered to ICR mice by intranasal or intravenous administration. 12 h later, AMs were collected and stained with pHrodo™ Red dextran for endosomal pH analysis. The data represent mean ± SD. * *p*<0.05, ** *p*<0.01, *** *p*<0.001, by two-tailed Student’s *t*-test (**a**) or by one-way ANOVA (**c-k, o-r**).

**Materials and Methods**

**Animals and Cell lines**

Female ICR mice were purchased from the Center of Medical Experimental Animals of the Chinese Academy of Medical Science (Beijing, China). Animals studies involving SARS-CoV-2 Delta were performed in an animal biosafety level 3 (BASL3) facility using HEPA-filtered isolators and the procedures were approved by the Institutional Animal Care and Use Committee of the Institute of Laboratory Animal Science, Peking Union Medical College (BLL20001). Human alveolar basal epithelial carcinoma cell line A549 was obtained from the Cell Resource Centre of Peking Union Medical College (Beijing, China) and cultured in DMEM medium (Gibco, Cat.: C11995500BT) with 10% FBS (Gibco, Cat.: 10099-141).

**Isolation of primary alveolar macrophages**

Primary alveolar macrophages (AMs) were isolated as described previously^1^. Briefly, the mice were anesthetized immediately prior to lavage and the trachea was dissected. Lungs were lavaged five times with 1 ml PBS and the retained BALF was centrifuged at 600 × g for 5 min at 4 °C. The pellet was resuspended in RPMI 1640 and harvested in a culture plate. The cells without stimulation are considered in a M0 state. M1 AMs were induced by LPS (100 ng/mL, Sigma, Cat. L2630) and IFN-γ (20 ng/mL, PeproTech, Cat. 315-05) or M2 were generated by IL-4 (20 ng/mL, PeproTech, Cat. 214-14).

**Stably overexpressing ACE2 in A549 cell line**

The human ACE2 coding sequence was amplified and inserted into a lentiviral vector plasmid pLV-EF1α-IRES-Puro (Addgene, Cat. 85132) for transient expression in 293T cells to obtain the virus containing the target gene. A549 cells transduced with lentiviruses containing hACE2 were selected with 1 μg /ml puromycin to obtain A549-ACE2 over-expressing cell clones.

**Virus infection *in vitro***

For virus infection with AMs, Delta or Omicron (2.5 × 10^4^ TCID_50_) was incubated with AMs (5 × 10^4^) for 2 h, then virus was removed and cells were re-cultured with dexamethasone (MCE, Cat: HY-14648, 0.2 μM or 1 μM), alendronate (Selleck, Cat: S1624, 10 μM or 50 μM) or combination for another 22 h or 46 h. For virus infection with ACE2-overexpression A549 cells, Delta (2.5 × 10^4^ TCID_50_) was incubated with ACE2-overexpression A549 cells (5 × 10^4^) for 4 h, then virus was removed and cells were re-cultured with dexamethasone (1 μM), alendronate (50 μM) or combination for another 20 h.

**Proteolytic reactions *in vitro***

Recombinant 2019-nCoV (Omicron, B.1.1.529) S-trimer Protein (DRA193), Recombinant 2019-nCoV (Delta, B.1.617.2) S-trimer Protein (DRA168), Recombinant 2019-nCoV (WT) S-trimer Protein (DRA49), and cathepsin L (C401) were purchased from Novoprotein. To assay cleavage of spike by cathepsin L in vitro, 1 μg purified spike protein (wild-type, Delta, Omicron) were incubated with 20 ng cathepsin L at 37 °C for 5 min. The reactions were carried out in a buffer containing 50 mM MES (M8010, Solarbio, China), 5 mM DTT (D8220, Solarbio, China), 1 mM EDTA (E8040, Solarbio, China) adjusted to pH 6.0 or 6.5. Following the incubation, 5 μL 6 × SDS sample loading buffer was added to the reaction mixture and heated to 95 °C for 5 min. The samples were detected by silver staining (Beyotime Biotechnology, P0017S, China).

**Real-time PCR**

Total RNA was extracted from cells using Trizol (Invitrogen) and was transcribed to cDNA by using a high capacity cDNA reverse transcription kit (Applied Biosystems, CA). The primer sequences are shown as follows: *Gapdh,* 5’- AGGTCGGTGTGAACGGATTTG-3’ (sense) and 5’-TGTAGACCATGTAGTTGAGGTCA-3’ (antisense); SARS-CoV-2 primer1 (*ORF1ab*): 5’-CCCTGTGGGTTTTACACTTAA-3’ (sense) and 5’-ACGATTGTGCATCAGCTGA-3’ (antisense); SARS-CoV-2 primer2 (*N*): 5’-GGGGAACTTCTCCTGCTAGAAT-3’ (sense) and 5’-CAGACATTTTGCTCTCAAGCTG-3’ (antisense); *TNF-α*, 5’-CCACGTCGTAGCAACCAC-3’ (sense) and 5’-TTGTCCCTTGAAGAGAACCTG-3’ (antisense); *IL-1β*, 5’- GCAACTGTTCCTGAACTCAACT -3’ (sense) and 5’- ATCTTTTGGGGTCCGTCAACT -3’ (antisense); *IL-6*, 5’-TAGTCCTTCCTACCCCAATTTCC -3’ (sense) and 5’- TTGGTCCTTAGCCACTCCTTC -3’ (antisense); C*tsl*, 5’-ATCAAACCTTTAgTgCAgAgTgg-3’ (sense) and 5’-CTgTATTCCCCgTTgTgTAgC-3’ (antisense); GAPDH, 5’- TGTGGGCATCAATGGATTTGG-3’ (sense) and 5’-CACCATGTATTCCGGGTCAAT-3’ (antisense).

**Immunofluorescence staining**

Cells were fixed and permeabilized for 5 min with 0.2% Triton X-100. After washed with PBS, cells were blocked with 5% BSA and incubated with anti-NP antibody (Abcam, Cat. Ab273434, 1:200), anti-Rab7 antibody (Abcam, Cat. ab137029, 1:200) or anti-CTSL antibody (ThermoFisher, Cat. MA1-26774, 1：400) at 4 ℃ overnight. Subsequently, cells were washed and incubated with secondary antibody for 1 h. Finally, cells were counterstained with DAPI and mounted for confocal analysis.

For lung tissues, the sections of paraffin embedded tissues were incubated with anti-Rab7 antibody (Abcam, Cat. ab137029, 1:200), anti-CTSL antibody (ThermoFisher, Cat. MA1-26774, 1：400) or anti-CD11c (1:200, CST, Cat. 97585S) antibody.

**Histological and immunohistochemical staining**

The lung tissues from mice were fixed in 10% formalin, embedded in paraffin and sectioned for H&E staining. The pathological score was acquired as previously described^1^. Immunohistochemical staining was performed according to a protocol as previously described^2^. In brief, the sections of paraffin embedded tissues were incubated with anti-TNF-α (1:200, Abcam, Cat. ab183218), anti-IL-6 (1:200, Abcam, Cat. ab208113) or anti-NP (1:500, Abcam, Cat. ab273434) antibody at 4 ℃ overnight. Afterwards, slides were sequentially incubated with HRP-conjugated secondary antibodies for 1 hr at room temprature. DAB (ZSGB-BIO, Cat: ZLI-9019) was used to react with HRP and hematoxylin was stained for cell nucleus. The stained lung sections were scanned and digitalized utilizing a TissueFaxs Plus System coupled onto a Zeiss Axio Imager Z2 microscope.

**Endosomal acidity detection**

For detecting the endosomal acidity, pHrodoTM red dextran (Thermo Fisher Scientific, USA) were utilized, which possesses a pH-sensitive fluorescent emission that increases in intensity with increasing acidity and is essentially non-fluorescent in the extracellular environment. Following the manufacturer’s guidelines, AMs were cultured with 50 μg/ml pHrodoTM red dextran in Live Cell Imaging Solution for 10 min at 37 ℃. After washing with pre-warmed medium, the cells were imaged by a Nikon A1 confocal microscope with appropriate filter or by Life Technologies Attune NxT.

**ELISA**

IL-1β (Thermo Fisher Scientific, Cat. BMS6002), TNF-α (Dakewei, Cat. 1217202) and IL-6 (Dakewei, Cat. 1210602) in BALF were quantified by ELISA according to the manufacturer’s protocol.

**Animal experiments**

To verify ALN and Dex whether affect CTSL and endosomal pH or not *in vivo*, female ICR mice (n = 3 per group) were intranasally infected with Dex, ALN, or both in a total volume of 50 μL. 24 hours later, AMs were isolated as described previously. To detect the anti-inflammation efficacy of drugs, LPS (Sigma, Cat: L2630, 100 μg / per mouse) was used to treat mice intranasally. Four hours later, Dex (1 μg / per mouse), ALN (150 μg / per mouse) or both were given. At 24 h, AMs were isolated as described previously and the BALF was collected.

To assess the efficacy of drugs in a realistic status, the hACE2 transgenic mouse model used is originally established from Chuan Qin’s lab^3^. hACE2 mice were infected with SARS-CoV-2 (1×10^5^ TCID_50_) by intranasal administration, 2 hours later, treated with vehicle control (PBS), Dex (i.n., 50 μl, 1 μg / per mouse), ALN (i.n., 50 μl, 150 μg / per mouse) or both once a day for 5 days. For intranasal administration, mice were anesthetized, and then ALN, Dex or both in a total volume of 50 μl was slowly delivered to nasal cavity with pipette. After 5 days of treatment, mice were euthanized and lung tissues were collected for real-time PCR assay and histological and immunohistochemical staining.

**Statistical Analysis**

All experiments were performed at least three times. Results are expressed as mean ± SD as indicated and analyzed by one-way ANOVA followed by Bonferroni’s test. P< 0.05 was considered statistically significant. The analysis was conducted using the Graphpad 8.0 software.

**Supplementary references**

1. Lv, J. et al. *Cell Discov* **7**, 24 (2021).

2. Wagner, J. et al. *Cell* **177** (2019).

3. Bao, L. et al. *Nature* **583**, 830-833 (2020).
